# Associations of depressive symptoms and chronic diseases with activities of daily living among middle-aged and older population in China: A population-based cohort study

**DOI:** 10.3389/fpsyt.2022.848255

**Published:** 2022-08-08

**Authors:** Yingyun Hu, Feixiang Zhou, Atipatsa Chiwanda Kaminga, Shipeng Yan, Zhao Hu

**Affiliations:** ^1^Hunan Cancer Hospital/The Affiliated Cancer Hospital of Xiangya School of Medicine, Central South University, Changsha, China; ^2^Department of Social Medicine and Health Management, Xiangya School of Public Health, Central South University, Changsha, China; ^3^Department of Mathematics and Statistics, Mzuzu University, Mzuzu, Malawi; ^4^Department of Epidemiology and Health Statistics, Xiangya School of Public Health, Central South University, Changsha, China

**Keywords:** chronic diseases, depressive symptoms, activities of daily living, cohort study, the elderly

## Abstract

**Background:**

Activities of daily living (ADL) disability is a concern in the aging population and can lead to increased health service demands and lower quality of life. The aim of this longitudinal study was to assess the associations of chronic conditions and depressive symptoms with ADL disability.

**Methods:**

This prospective cohort study used two waves of data (2011 and 2015) from the China Health and Retirement Longitudinal Study (CHARLS). A total of 10,864 participants aged 45 and older were included for analysis. Chronic diseases were assessed by self-report and depressive symptoms were assessed using the validated 10-item of Center for Epidemiologic Studies Depression Scale at baseline. Incidents of ADL disability during follow-up were assessed using the Katz ADL scales.

**Results:**

After 4 years of follow-up, there were 704 participants incidents of ADL disability. The incident rate was 17.22 per 1,000 person-years. Having at least one chronic disease was independently associated with a 39% increased risk of incident ADL disability (adjusted HR, 1.39; 95%CI: 1.16, 1.67). The presence of depression symptoms was independently associated with a 54% increased risk of incident ADL disability (adjusted HR, 1.54; 95%CI: 1.30, 1.82). However, there was no significant additive interaction effect between chronic diseases and depressive symptoms on ADL disability.

**Conclusion:**

Chronic diseases and depressive symptoms are associated with an increased risk of ADL disability in middle-aged and older Chinese adults. Improving chronic diseases and depressive symptoms can prevent ADL disability.

## Introduction

The increasing trend of the aging population is predictable all over the world, and China's aging population is estimated to increase at the rate of 6.2 million per year from 2021 to 2050, and is expected to exceed 400 million by 2050 (1). An aging population raises many concerns in the domain of health. For example, the impact of functional disability on individuals, especially in middle-aged and older adults, is manifested by increased demand for health services, reduced subjective wellbeing, and increased risk of death (2–4). Not only China, other East Asian countries, facing huge challenges. For example, the prevalence of functional disability in an elderly general population of Japanese was 20.1 and 4.9% of elderly in Korea reported functional decline of at least one of the seven activities of daily living (ADL) components after 2 years (5, 6). Moreover, the prevalence of depression was 33.5% in elderly Japanese and subjects with depression revealed significantly lower scores for ADL than those without depression (7).

Life expectancy is increasing worldwide, at the same time people are living longer with disabilities as well as functional health loss (8). ADL disability is the main source of functional disability, which are activities that individuals hard to perform to manage their basic physical needs in order to live independently and need help from others (9). The physical frailty indicators, such as body weight, gait speed, physical activity, and balance are predictors of future ADL disability (10, 11). ADL disability does not directly lead to death, but affects the quality of life, the burden of care and the prognosis of other diseases. Participants with ADL disability tended to have a lower quality of life based on the evidence that functional disability was significantly associated with all dimension scores of the 36-item short form survey (SF-36) in a cross-sectional study (12). According to a prospective study of 1,521 elderly individuals, ADL disability was a significant factor in increased use of care as reflected in the fact that medical expenditures were 2–3 times higher for patients with moderate to severe functional disabilities in the outpatient, emergency, and inpatient services (13).

Several studies from various regions have reported associations of chronic diseases or depressive symptoms with ADL disability but the magnitude of association remains to be assessed (14–17). According to the Canadian Study of Health and Aging (CSHA), among 9,008 community residents aged 65 and older, the occurrence of chronic diseases accounted for about 66% of the ADL disability (15). These findings suggest that chronic diseases are potential risk factors for functional disability, but evidence from cohort studies and the magnitude of association is lacking. Meanwhile, the effect of depressive symptoms on ADL disability is worthy of attention. People with depressive symptoms had a 130% greater risk of ADL disability than those without depressive symptoms among the African Americans of the Jackson Heart Study (16). In the Community Early Psychiatric Intervention Strategies (CEPIS) program in Singapore, higher levels of depressive symptoms were associated with a higher risk of ADL disability (17). However, the lack of evidence from large samples in China makes it impossible to assess the heterogeneity among current studies.

We aimed to explore the associations of chronic diseases and depressive symptoms with ADL disability according to the data from the China Health and Retirement Longitudinal Study (CHARLS). This has the potential benefit of providing a basis for targeted prevention policies to improve the efficiency of health services and reduce the burden of care.

## Materials and methods

### Study population

Participants in this cohort study were from the China Health and Retirement Longitudinal Study (CHARLS), which is an ongoing nationally representative longitudinal study. Details of the study design have been described elsewhere (18). In brief, a total of 17,708 participants were recruited by a multistage probability sampling procedure involving 150 counties or districts and 450 communities within 28 provinces of China at baseline in 2011 with a response rate of 80.5%. Data on socio-demographic and lifestyle factors and health-related information were collected by about 500 professional interviewers who worked in this field. Quality of these data was checked using a computer-assisted personal interviewing system. All participants were followed up biennially after the baseline survey. Therefore, this study used participants from two waves of the CHARLS (2011 and 2015), who were aged 45 and above at baseline. The CHARLS was approved by the Biomedical Ethics Review Committee (IRB00001052-11015), and written informed consent was obtained from all participants. At baseline, 17,708 CHARLS participants were enrolled. Of these, 423 individuals were excluded for being younger than 45 years, and 2,379 were excluded for having any disabilities at baseline. Further, respondents who had missing values on depressive symptoms, chronic diseases or covariates were excluded (*n* = 4,042). Finally, 10,864 participants were included for analysis in this study.

### Assessment of chronic diseases

Chronic diseases were self-reported based on a physician's diagnosis using the following standardized question: “Have you been diagnosed with such conditions as listed below by a doctor?.” In this regard, CHARLS provided 14 chronic diseases in the inventory as follows: hypertension, dyslipidemia, diabetes, cancer or malignant tumor, chronic lung diseases (such as chronic bronchitis and emphysema), liver disease, heart problems (such as heart attack, coronary heart disease, angina, and congestive heart failure), stroke, kidney disease, stomach or other digestive diseases, emotional, nervous, or psychiatric problems, memory-related disease (such as dementia, brain atrophy, and Parkinson's disease), arthritis or rheumatism and asthma. Each of the conditions was ascertained one by one by the interviewers.

### Assessment of depressive symptoms

Depressive symptoms in the past week were assessed at baseline using the 10-items Center for Epidemiologic Studies Depression (CESD-10) scale short form (19, 20). Each item was rated on a 4-point Likert scale with answers varying from 0 [rarely or none of the time (<1 day)] to 3[most or all of the time (5–7 days)]. The items 5 and 8 were reverse scored before summing all the item scores. The total score of CESD-10 ranged from 0 to 30, and higher scores indicated more depressive symptoms. The CESD-10 has been widely used in epidemiological studies and has high reliability and validity among Chinese adults (21). According to previous studies, participants with a total score of 12 or higher were defined as having elevated depressive symptoms (21).

### Outcome ascertainment

The study outcome was incident ADL functional limitations. Functional limitations at follow-up were assessed using the Katz ADL scales (22). The Katz ADL scale assessed daily self-care tasks including taking bathing, dressing, eating, getting into and out of bed, toileting and controlling urination and defecation. The Katz ADL scale was extensively used in previous studies among Chinese older adults, and the Chinese version provided reliable and valid responses (23, 24). Each of the following answers, as provided in CHARLS, was used to assess each of the preceding items in the Katz ADL scale: (1) No, I do not have any difficulty, (2) I have difficulty but still can do it, (3) Yes, I have difficulty and need help, and (4) I cannot do it. Participants who reported needing any help in any item were classified as having ADL disability (25). Participants who reported one or more limitations during the follow-up period were defined as having incident ADL disability.

### The measures of covariates

The socio-demographic information included age, gender, marital status and education level. Marital status was divided into married and non-married. That is, non-married status included the separated, divorced, widowed, and never married. Education level was classified as no formal education, primary school, middle or high school, and college or above.

Health-related information included self-rated childhood health, history of fall (yes/no), history of hip fracture (yes/no), history of traffic accident (yes/no), smoking (current/ex-smoker and no), alcohol drinking (often/seldom/never), sleep duration at night (<7, 7–8, and >8 h), nap duration after lunch (<30, 30–60, and >60 min), and body pain (yes/no). All these covariates were associated with ADL disability in the elderly in previous studies (26, 27). Self-rated childhood health status was assessed by asking participants the following question: “how would you evaluate your health during childhood, up to and including the age of 15?.” In this regard, the participants were asked to rate their health on a five points scale with the following response categories: (5) excellent, (4) very good, (3) good, (2) fair, and (1) poor. Further, (5), (4), and (3) were combined to indicate “good health.”

Participants who drank alcohol (such as beer, wine, or liquor) more than once a month in the past year were defined as often drinkers. Sleep duration at night was self-reported according to the question: “during the past month, how many hours of actual sleep did you get at night?” Sleep duration was divided into three groups: <7, 7–8, and >8 h. Nap duration was measured using a single question as follows: “during the past month, how long did you take a nap after lunch?”. Nap duration was classified into three groups: <30, 30–60, and >60 min. History of fall was assessed using a single item: “have you fallen down in the last two years?.” Body pain was assessed by the question: “are you often troubled with any body pains?.”

Social participation was measured by asking respondents to indicate whether they have participated in various social activities including playing mah-jong, and chess or cards; attending a community, sports, and social or other clubs; participating in a community-related organization; participating in voluntary or charity work; and attending any educational or training courses in the past 12 months. Participants who have not engaged in any activities stated above were defined as low social participation.

### Statistical analysis

Data were summarized as mean and standard deviation (*SD*) for each normally distributed continuous variable, and as median and interquartile range for each non-normally distributed continuous variable. Frequencies and percentages were used to describe categorical variables. Baseline characteristics were summarized and stratified according to the depression symptoms. We used the Chi-square test, Student's *t* test or Mann-Whitney *U* test to examine the differences in sample characteristics between those with and without depression symptoms.

Person-time of follow-up for each subject from the date of 2011 to the dates of ADL disability ascertainment (every 2 years), death, loss to follow-up, or the end of follow-up (June 31, 2015), whichever came first. The incidence rate of ADL disability per 1,000 person-years was calculated based on cases of ADL disability and total person-time of follow-up according to different status of chronic disease (yes/no) and depression symptoms (yes/no), respectively. The association between chronic diseases, depressive symptoms and ADL disability was examined using Cox proportional hazards models, from which hazard ratios (HRs) with their corresponding 95% confidence intervals (CI) were obtained. Three models were estimated: in model 1, age and gender were adjusted; in model 2, age, gender, education, marital status, smoking, alcohol drinking, sleep duration, and nap duration were adjusted; and in model 3, variables in model 2 plus childhood health status, history of fall, history of hip fracture, history of traffic accident, body pain, and social participation were adjusted.

To further examine the association between the severity of depression symptoms and risk of ADL disability, the scores of depressive symptoms were split into quintiles and then were included in Cox proportional hazards models with quintile 1 as the reference group. Additionally, we explored the potential non-linear associations using 5-knotted restricted cubic spline regression.

To further examine the potential additive interaction between chronic diseases and depressive symptoms on the risk of incident ADL disability, four subgroups were created as follows: (1) people with no chronic disease and depressive symptoms, (2) people with no chronic disease but have depressive symptoms, (3) people with at least one chronic disease but no depressive symptoms, and (4) people with at least one chronic disease and depressive symptoms. Group (1) was the reference group. Synergy Index (SI) was used to provide more insight on the additive interaction between them (28). The synergy index is the ratio of the risk of the joint effect to the sum of the individual risks. If the confidence interval of SI contains 1, it indicates that the two factors have no interaction (29). All analyses were performed using STATA statistical software version 16.0 (STATA Corp, College Station, Texas, USA). Two-sided *P* < 0.05 was considered statistically significant.

## Results

### Baseline information

Baseline characteristics of participants according to depressive symptoms status are presented in Table 1. Thus, the mean (*SD*) age at baseline was 58.1 (8.9) years. In addition, 5,336 (49.1%) of the participants were men, 2,577 (23.7%) had no formal education and 1,154 (10.6%) of them had unstable marital status. A total of 2,608 (24.0%) of the participants reported having depressive symptoms. Furthermore, 6,520 (60.0%) of the participants never smoked, 2,882 (26.5%) often drank alcohol, 1,511 (13.9%) had a history of fall down, 1,053 (9.7%) had a history of traffic accidents and 133 (1.2%) had a history of hip fracture. Regarding chronic diseases, 7,078 (65.2%) individuals reported having at least one chronic disease.

**Table 1:** Baseline characteristics of participants according to depressive symptoms status.

**Characteristics**	**Total sample** **(*****n*** = **10,864)**	**Depressive symptoms**		*P* ^a^
		**Yes (*****n*** = **2,608)**	**No (8,256)**	
Age, mean (*SD*), y	58.1 (8.9)	59.0 (8.8)	57.8 (8.9)	<0.001
Men	5,336 (49.1)	989 (37.9)	4,347 (52.7)	<0.001
**Education level**				
No formal	2,577 (23.7)	807 (30.9)	1,770 (21.4)	<0.001
Primary school	4,447 (40.9)	1,205 (46.2)	3,242 (39.3)	
Middle or high school	3,600 (33.1)	577 (22.1)	3,023 (36.6)	
College or above	240 (2.3)	19 (0.7)	221 (2.7)	
Non-married	1,154 (10.6)	430 (16.5)	724 (8.8)	<0.001
**Smoking**				
Never	6,520 (60.0)	1,710 (65.6)	4,810 (58.3)	<0.001
Former	882 (8.1)	174 (6.7)	708 (8.6)	
Current	3,462 (31.9)	724 (27.8)	2,738 (33.2)	
**Alcohol drinking**				
Often	2,882 (26.5)	560 (21.5)	2,332 (28.1)	<0.001
Seldom	866 (8.0)	176 (6.7)	690 (8.4)	
No	7,116 (65.5)	1,872 (71.8)	5,244 (63.5)	
**Childhood health status**				
Good	8,277 (76.2)	1,803 (69.1)	6,474 (78.4)	<0.001
Fair	1,886 (17.4)	552 (21.2)	1,334 (16.2)	
Poor	701 (6.5)	253 (9.7)	448 (5.4)	
Body pain	3,163 (29.1)	1,448 (55.5)	1,715 (20.8)	<0.001
History of fall	1,511 (13.9)	545 (20.9)	966 (11.7)	<0.001
History of traffic accident	1,053 (9.7)	308 (11.8)	745 (9.0)	<0.001
History of hip fracture	133 (1.2)	46 (1.8)	87 (1.1)	0.004
**Sleep duration**				
<7 h	5,342 (49.2)	1,690 (64.8)	3,652 (44.2)	<0.001
7–8 h	4,658 (42.9)	764 (29.3)	3,894 (47.2)	
>8 h	864 (8.0)	154 (5.9)	710 (8.6)	
**Nap duration**				
<30 min	5,268 (48.5)	1,427 (54.7)	3,841 (46.5)	<0.001
30–60 min	3,746 (34.5)	805 (30.9)	2,941 (35.6)	
>60 min	1,850 (17.0)	376 (14.4)	1,474 (17.9)	
Low social participation	5,193 (47.8)	1,431 (54.9)	3,762 (45.6)	<0.001
Number of chronic diseases^b^, median (lower-upper quartile)	1.0 (0.0–2.0)	1.0 (0.0–2.0)	1.0 (1.0–3.0)	<0.001
At least one chronic disease	7,078 (65.2)	2,004 (76.8)	5,074 (61.5)	<0.001
Hypertension	2,415 (22.2)	625 (24.0)	1,790 (21.7)	0.014
Diabetes	536 (4.9)	145 (5.6)	391 (4.7)	0.09
Dyslipidemia	927 (8.5)	248 (9.5)	679 (8.2)	0.041
Cancer or malignant tumor	95 (0.9)	36 (1.4)	59 (0.7)	0.001
Chronic lung diseases	971 (8.9)	343 (13,2)	628 (7.6)	<0.001
Liver diseases	400 (3.7)	126 (4.8)	274 (3.3)	<0.001
Heart problems	1,154 (10.6)	395 (15.1)	759 (9.2)	<0.001
Stroke	145 (1.3)	48 (1.8)	97 (1.2)	0.01
Kidney disease	623 (5.7)	238 (9.1)	385 (4.7)	<0.001
Stomach or other digestive disease	2,371 (21.8)	805 (30.9)	1,566 (19.0)	<0.001
ENP	95 (0.9)	55 (2.1)	40 (0.5)	<0.001
Memory-related disease	88 (0.8)	26 (1.0)	62 (0.8)	0.222
AR	3,504 (32.3)	1,240 (47.5)	2,264 (27.4)	<0.001
Asthma	313 (2.9)	126 (4.8)	187 (2.3)	<0.001

Data were summarized as mean (SD) or median (lower-upper quartile) or n (%).

a P-value was based on Chi-square test or student's t test or Mann-Whitney U test where appropriate.

b Number of chronic diseases was the total number of chronic diseases each subject reported, which ranged from 0 to 14.

ENP, emotional, nervous, or psychiatric problems; AR, Arthritis or rheumatism.

Arthritis or rheumatism (32.3%) was the most reported chronic disease in the middle-aged and older population. This was followed by hypertension (22.2%), then stomach or other digestive diseases (21.8%). Diabetes was reported in 536 (4.9%) of the participants, and 927 (8.5%) reported having dyslipidemia. Participants with the following attributes were more likely to have depressive symptoms: older, women, not married, lower education level, never smoking and drinking, less sleep and nap duration, poor self-rated childhood health, a history of fall, traffic accident or hip fracture, low social participation, a higher prevalence of hypertension, dyslipidemia, cancer, chronic lung disease, liver disease, digestive disease, heart problems, stroke, kidney disease, ENP, AR, and asthma.

### The associations between chronic disease, depressive symptoms and ADL disability

During the follow-up period from 2011 to 2015, a total of 704 participants reported having incident ADL disability. The incident rate of ADL disability was 17.22 per 1,000 person-years. The incident rate was 11.29 per 1,000 person-years among participants without chronic disease and 20.40 per 1,000 person-years among participants with chronic disease. The incident rate was 13.70 per 1,000 person-years among participants without depression symptoms and 28.48 per 1,000 person-years among participants with depression symptoms.

**Figure 1:**
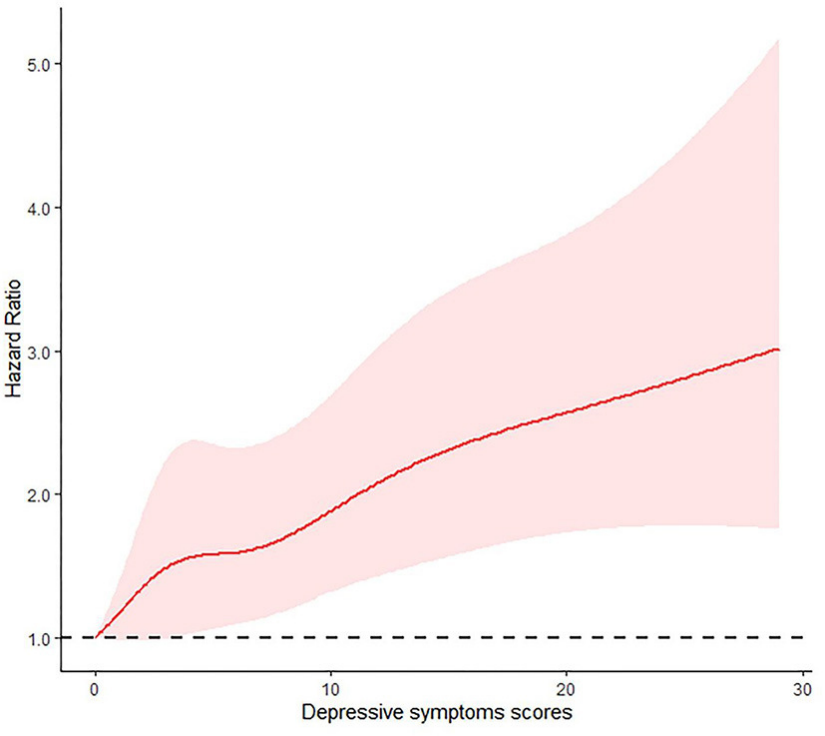
Adjusted Hazard Ratios (HRs) of depressive symptoms scores on ADL disability. Results from the restricted cubic spline Cox proportional hazards regression model.

Table 2 shows the associations between chronic disease, depressive symptoms and ADL disability. After adjusting for potential confounders in model 3, the results indicated that having at least one chronic disease among participants was independently associated with an increased risk of ADL disability (adjusted HR, 1.39; 95%CI: 1.16–1.67). Specifically, hypertension, diabetes, chronic lung diseases, heart problems, stroke, arthritis or rheumatism, and asthma were associated with the increased risk of ADL disability after adjusting for potential confounders. Moreover, participants with depressive symptoms were more likely to report ADL disability after adjusting for potential confounders (adjusted HR, 1.54; 95%CI: 1.30–1.82). Similar results were obtained when modeling the total CESD-10 scores as quintiles (Table 2). After adjusting for confounders, by comparing quintile 5 score with quintile 1, the adjusted HR was 2.03(95%CI: 1.54–2.57) for incident ADL disability. A linear and positive association between the CESD-10 total score and risk of incident ADL events using restricted cubic spline regression was also found (for non-linearity, *P* = 0.383) (Figure 1).

**Table 2:** Association between chronic diseases, depression symptoms and ADL disability.

**Variables**	**Case, No**	**Incident rate, per** **1,000 person- years**	**HR (95%CI)**		
			**Model 1** ^a^	**Model 2** ^b^	**Model 3** ^c^
**At least one chronic diseases**					
No	161	11.29	Reference	Reference	Reference
Yes	543	20.40	1.59 (1.33, 1.90)	1.55 (1.30, 1.85)	1.39 (1.16, 1.67)
**Each of chronic diseases**					
Hypertension	218	23.98	1.30 (1.11, 1.53)	1.30 (1.11, 1.53)	1.26 (1.09, 1.49)
Dyslipidemia	74	21.54	1.22 (0.95, 1.55)	1.27 (0.99, 1.62)	1.18 (0.93, 1.51)
Diabetes	57	28.53	1.59 (1.21, 2.08)	1.61 (1.23, 2.11)	1.52 (1.16, 2.00)
Cancer or malignant tumor	7	19.89	1.09 (0.52, 2.30)	1.04 (0.49, 2.18)	1.00 (0.47, 2.09)
Chronic lung diseases	100	27.61	1.55 (1.26, 1.92)	1.49 (1.20, 1.84)	1.37 (1.11, 1.71)
Liver diseases	33	21.68	1.37 (0.96, 1.94)	1.39 (0.98, 1.98)	1.26 (0.89, 1.79)
Heart problems	114	26.09	1.34 (1.10, 1.64)	1.35 (1.10, 1.65)	1.24 (1.01, 1.53)
Stroke	59	25.19	1.56 (1.19, 2.04)	1.54 (1.18, 2.02)	1.34 (1.02, 1.75)
Kidney disease	15	27.68	1.33 (0.80, 2.22)	1.34 (0.80, 2.23)	1.19 (0.71, 1.99)
Stomach or other digestive disease	181	20.27	1.27 (1.07, 1.50)	1.23 (1.04, 1.46)	1.10 (0.92, 1.31)
ENP	11	25.22	1.67 (0.92, 3.02)	1.56 (0.86, 2.83)	1.32 (0.73, 2.41)
Memory-related disease	15	30.18	1.84 (1.10, 3.08)	1.89 (1.13, 3.17)	1.68 (0.99, 2.81)
AR	325	24.85	1.69 (1.46, 1.96)	1.64 (1.41, 1.89)	1.45 (1.25, 1.69)
Asthma	39	23.85	1.63 (1.18, 2.26)	1.59 (1.15, 2.20)	1.42 (1.03, 1.97)
**Depressive symptoms**					
No	427	13.70	Reference	Reference	Reference
Yes	277	28.48	1.86 (1.60, 2.17)	1.76 (1.51, 2.06)	1.54 (1.30, 1.82)
**Depressive symptoms scores, quintile**					
1 [0–3]	120	10.54	Reference	Reference	Reference
2 [4–6]	121	14.08	1.30 (1.01, 1.68)	1.26 (0.98, 1.63)	1.22 (0.96, 1.55)
3 [7–9]	116	16.91	1.49 (1.15, 1.92)	1.43 (1.11, 1.85)	1.34 (1.03, 1.74)
4 [10–14]	166	22.06	1.92 (1.52, 2.43)	1.80 (1.41, 2.28)	1.58 (1.24, 2.05)
5 [15–30]	181	31.47	2.66 (2.11, 3.36)	2.47 (1.94, 3.13)	2.03 (1.54, 2.57)

HR, hazard ratio; CI, confidence interval. ENP, emotional, nervous, or psychiatric problems; AR, Arthritis or rheumatism.

a Model 1 adjusted for age and gender.

b Model 2 plus education level, marital status, smoking, alcohol drinking, sleep duration, nap duration

c Model 3 plus childhood health status, history of fall, history of hip fracture, history of traffic accident, body pain and social participation.

The incidence rate was 10.00 per 1,000 person-years among participants without chronic disease and depression symptoms. The incidence rate was 31.62 per 1,000 person-years among participants with both chronic disease and depression symptoms. However, there is no statistically significant additive interaction between chronic disease and depression symptoms for incident ADL disability. The Synergy Index was 1.34 (95%CI: 0.65–2.75). The result was presented in Table 3.

**Table 3:** The interaction between chronic diseases and depressive symptoms on incident ADL disability.

**Chronic diseases**	**Depressive symptoms**	**Case, No**	**Incident rate, per** **1,000 person-years**	**ADL disability**^a^ **HR (95%CI)**
No	No	120	10.00	Reference
No	Yes	41	18.12	1.43 (1.00, 2.05)
Yes	No	307	16.02	1.34 (1.08, 1.65)
Yes	Yes	236	31.62	2.02 (1.59, 2.57)
Synergy Index (95%CI)			1.34 (0.65, 2.75)	

ADL, activities of daily living; HR, hazard ratio; CI, confidence interval.

a Adjusted for age, gender, education level, marital status, smoking, alcohol drinking, sleep duration, nap duration, childhood health status, history of fall, history of hip fracture, history of traffic accident, body pain and social participation.

## Discussion

This study has shown that chronic diseases and depressive symptoms were significantly associated with ADL disability. That is, people with at least one chronic disease had a 39% higher risk of developing functional disability than those without chronic disease. Also, depressive symptoms were positively associated with the risk of ADL disability.

The data from the longitudinal study were analyzed to elucidate the role of chronic diseases and depressive symptoms in the pathway from normal to ADL disability. Our study increased the evidence for the associations of chronic diseases and depressive symptoms with risk of ADL disability. In total, we considered 14 types of chronic diseases and estimated the magnitude of association between chronic diseases and ADL disability. According to a previous population-based cross-sectional study, ADL disability after a recent stroke was associated with a 45% higher risk of recurrent stroke (30). Stroke was the most common cause of ADL disability in subjects aged 65–74 years (6). Physical disability was highest for stroke, followed by cancer and diabetes at the time of the initial disease diagnosis (31). Diabetic polyneuropathy (DPN) is a common complication of diabetes, and the symptom of decreased muscle strength occurs in the lower limbs and progresses upward, eventually emerging in the upper extremities (32). Muscle strength is an important factor in maintaining ADL (33).

Consistent with the findings of this study, depression symptoms was strongly associated with ADL disability, even after adjustment for demographic factors, socioeconomic conditions, and chronic diseases (34, 35). The finding of the Chicago Neighborhood and Disability Study showed that depressive symptoms were associated with ADL disability in both older blacks and whites. Meanwhile, depressive symptoms increased the odds of onset of disability over time in whites more than blacks (36). Our study suggests that the improvement of depressive symptoms has a positive preventive effect on ADL disability in Chinese. However, several studies demonstrated that people with ADL dependency would be more likely to develop depressive symptoms (37, 38). The relationships between each other need more studies to explore in the future.

Inflammation and albuminuria are involved in the effects of chronic diseases on ADL disability, and the detection of these biomarkers has clinical significance. Elevated levels of C-reactive protein (CRP) and urinary albumin-to-creatinine ratio were independently associated with ADL disability among older adults with cardiovascular disease, and subjects with higher levels of both markers had a more unfavorable metabolic profile than those with lower levels (39). In addition, macroalbuminuria was associated with disability in ADL (OR = 1.94, 95%CI: 1.24–3.03) and there was an interaction effect between elevated CRP and albuminuria on ADL disability among older adults with diabetes (40). Meanwhile, high-sensitivity CRP has sufficient value as a predictor of the prognosis of ADL disability after the first-ever stroke (41).

There are several potential mechanisms on the association between depressive symptoms and risk of ADL disability. First, this increased risk is partly due to the fact that people with depression decrease physical activity (42, 43). Physical activity is a beneficial protective factor of ADL disability that deserves attention. Second, depression is a risk factor for non-adherence to treatment that can affect prognosis and lead to poor outcomes, including ADL disability (44). Patients with comorbid depression and other diseases, such as diabetes and heart problems, have more symptoms of the disease, making it difficult to recover and leading to disability development (45). Considering this, policymakers should adopt strategies to improve chronic disease management in the community while screening older adults for mental health issues to prevent ADL disability.

Several limitations of this study need to be acknowledged. First, there are problems with the accuracy of self-reported depression symptoms and medical status in large-scale follow-up surveys, thus recall bias cannot avoidable. Second, it would be cautious to infer the causal relationship due to the observational study design. Third, only participants from China were involved in this study, thus the findings may not fully generalize to other countries and populations. Fourth, we did not adjust the analysis for the covariate such as financial situation and cognition, and they affected ADL and also affected depressive symptoms (46, 47), thus confounding bias could not be ruled out. Fifth, we did not find a statistically significant interaction between chronic diseases and depressive symptoms on incident ADL disability. However, people with chronic diseases and depressive symptoms have a higher risk of ADL disability based on the point estimate. Finally, our study provided evidence for previous studies and verified these results, however, an inherent limitation is lack of substantial novelty.

## Conclusions

Chronic diseases and depressive symptoms are associated with an increased risk of ADL disability in middle-aged and older Chinese adults. Potential strategies need to focus on improving chronic diseases and depressive symptoms to prevent functional disability in the future.

## Data availability statement

CHARLS data are available at http://charls.pku.edu.cn/pages/data/111/zh-cn.html.

## Ethics statement

The studies involving human participants were reviewed and approved by the Biomedical Ethics Review Committee of Peking University. Written informed consent to participate in this study was provided by the participants' legal guardian/next of kin.

## Author contributions

ZH designed the study. YH and FZ drafted the manuscript. ZH, ACK, and SY edited the manuscript. All authors approved the final version of the manuscript.

## Funding

This study was supported by the Natural Science Foundation of Hunan Province (No. 2022JJ40248), Hunan Cancer Hospital Climb Plan (No. QH2021003), and Scientific Research Project of Hunan Provincial Health Commission (No. 202212054721).

## Conflict of interest

The authors declare that the research was conducted in the absence of any commercial or financial relationships that could be construed as a potential conflict of interest.

## Publisher's note

All claims expressed in this article are solely those of the authors and do not necessarily represent those of their affiliated organizations, or those of the publisher, the editors and the reviewers. Any product that may be evaluated in this article, or claim that may be made by its manufacturer, is not guaranteed or endorsed by the publisher.
